# Oral Manifestations in Adolescents with Genetic Syndromes: A Retrospective Cross-Sectional Study

**DOI:** 10.3390/jcm14207217

**Published:** 2025-10-13

**Authors:** Adriana Țenț, Raluca Iurcov, Abel Emanuel Moca, Rahela Tabita Moca, Ioan Andrei Țig, Ruxandra Ilinca Matei

**Affiliations:** Department of Dentistry, Faculty of Medicine and Pharmacy, University of Oradea, 4 Universității Street, 410087 Oradea, Romania; adriana.tent@uoradea.ro (A.Ț.); raluirimie@yahoo.com (R.I.); rahelamoca@gmail.com (R.T.M.); itig@uoradea.ro (I.A.Ț.); rmatei@uoradea.ro (R.I.M.)

**Keywords:** genetic syndromes, pediatric dentistry, dental anomalies, hypodontia, enamel hypoplasia, taurodontism, phenotypic markers, adolescents

## Abstract

**Background/Objectives**: Few studies have comprehensively examined dental anomalies in adolescents with genetic syndromes. This study aimed to assess their prevalence, types, and clinical patterns in a diverse sample of genetically confirmed cases. **Methods**: We conducted a retrospective cross-sectional study of 213 patients aged 12 to 18 years with various genetic syndromes, using clinical data originally collected between 2011 and 2014 at a tertiary center. Clinical examinations were complemented by radiographs when available. Anomalies were categorized by type, and a disproportionality analysis (Rate of Occurrence Ratio, ROR) quantified risk relative to syndrome representation. **Results**: Dental anomalies were present in 68% of adolescents. The most common findings were hypodontia, taurodontism (9%), and enamel hypoplasia (8%). Nearly half of the patients exhibited combined patterns, with hypodontia–taurodontism as the most characteristic combination (14%). Prevalence was particularly high in trisomy-based (80%) and osteogenesis-related (100%) syndromes. Down syndrome showed the strongest association (ROR 3.95; 95% CI: 2.15–7.25), while some conditions such as Turner, Ehlers-Danlos, and Tuberous sclerosis displayed significantly lower rates. **Conclusions**: Dental anomalies are both highly prevalent and patterned in adolescents with genetic syndromes. Their co-occurrence and specificity suggest that they may serve as useful diagnostic markers in syndromic evaluation.

## 1. Introduction

Genetic syndromes frequently present with distinctive craniofacial and dental features, making oral examination a valuable adjunct to early diagnosis. It is estimated that approximately 900 of the more than 5000 known genetic syndromes (~18%) exhibit dento-oro-maxillofacial anomalies as part of their clinical phenotype, particularly those involving ectodermal derivatives [1]. These anomalies, ranging from minor morphological variations to extensive tooth agenesis, result from genetically determined processes or developmental disruptions and may appear in isolation or as part of broader syndromic contexts [2,3].

In the general population, developmental dental anomalies are relatively uncommon, with reported prevalence rates typically ranging from 5% to 10% depending on the anomaly and population studied. Hypodontia is the most frequent condition, affecting approximately 6–8% of non-syndromic permanent dentitions, while taurodontism and enamel hypoplasia occur in less than 5% of healthy cohorts [4,5,6]. These anomalies, although clinically relevant, are usually isolated findings. In contrast, syndromic patients often present with much higher frequencies and distinctive combinations of anomalies, reflecting the broader genetic disturbances underlying craniofacial and dental development [7,8,9].

Several syndromes are consistently associated with a high prevalence of dental anomalies. In Down syndrome (trisomy 21) abnormalities are reported in over 95% of affected individuals, most commonly hypodontia (particularly of lateral incisors and second premolars), microdontia, taurodontism, delayed eruption, and over-retained primary teeth [7,8,9]. Ectodermal dysplasia typically presents with severe oligodontia and conical teeth, whereas junctional forms of epidermolysis bullosa are more often associated with enamel hypoplasia resembling hypoplastic amelogenesis imperfecta. Cleidocranial dysplasia, by contrast, is characterized by multiple supernumerary and impacted teeth, sometimes preceding systemic manifestations and prompting genetic evaluation [10,11]. A recent study further confirmed that children with genodermatoses display significantly higher rates of enamel defects and dental anomalies than healthy controls [12].

Across these syndromes, the most common oral findings involve anomalies of tooth number, form, structure, and eruption. Such features are clinically significant not only as potential early diagnostic indicators, but also because they affect function, aesthetics, and quality of life, often requiring complex multidisciplinary care [7,8,9,10,11,12].

Despite these observations, the available evidence remains fragmented. Much of the current knowledge derives from isolated case reports, small single-syndrome cohorts, or heterogeneous methodologies, which limits the ability to establish prevalence patterns across broader syndromic populations. Regional data are particularly scarce in Eastern Europe. A previous Romanian study focused only on children under six years of age and reported dental anomalies in 79% of cases, most commonly enamel or dentin defects, eruption disturbances, and size anomalies [13]. However, findings in preschool-aged children cannot be extrapolated to older groups, as key conditions such as hypodontia, taurodontism, or complex eruption disturbances often become fully apparent only with the establishment of permanent dentition [14,15]. Adolescence is also the stage when these anomalies acquire the greatest clinical significance, given the increasing demand for orthodontic and restorative interventions [16]. Yet, systematic studies including larger samples of adolescents with genetically confirmed syndromes remain lacking.

To address this gap, the present study focuses specifically on adolescents (12–18 years) with genetically confirmed syndromes, a group for which regional data are virtually absent. Using standardized classification criteria and disproportionality analysis, this study provides the first systematic prevalence estimates for Romanian patients in this age range. By characterizing anomaly patterns across multiple syndromic diagnoses, the study seeks to generate clinically relevant phenotypic markers, contribute new epidemiological evidence for Eastern Europe, and underline the role of dental screening in early recognition and multidisciplinary management.

The specific aim of this study was to provide the first systematic analysis of dental anomalies in Romanian adolescents with genetically confirmed syndromes, a population for which no prevalence data currently exist. By applying standardized classification criteria and quantitative disproportionality analysis, we sought to expand upon previous Romanian findings limited to preschool children and to clarify how key anomalies, such as hypodontia, taurodontism, and eruption disturbances, manifest once permanent dentition is established. Focusing on adolescents is particularly relevant, as this stage combines diagnostic visibility with the greatest clinical demand for orthodontic and restorative care, thereby linking early developmental disturbances to long-term oral health needs.

## 2. Materials and Methods

### 2.1. Study Design and Population

This observational, cross-sectional study retrospectively analyzed clinical data from 213 children and adolescents aged 12–18 years, all of whom had confirmed diagnoses of genetic syndromes. The study was conducted in Oradea, a city in Bihor County, north-western Romania (Europe). Participants were identified through medical records from the Genetics Department of Dr. Gavril Curteanu Municipal Hospital, the main tertiary care provider for the region, and from two local special education institutions (Special School No. 2 and Auxiliary School No. 3). This geographical context is relevant, as the hospital serves as a referral center for genetic disorders in north-western Romania. The original data were collected between 2011 and 2014 as part of the first author’s doctoral research, conducted in a clinical setting. The dataset was reviewed, curated, and reanalyzed for the present study using updated diagnostic classifications and statistical methods. Diagnostic criteria were updated according to the 2019 framework by de La Dure-Molla et al. [17]. This did not alter the overall inclusion status, but several anomalies were reclassified within categories.

Inclusion criteria required a genetically confirmed diagnosis of a syndromic condition, age between 12 and 18 years, and availability of clinical dental documentation. Genetic confirmation was operationalised as the presence of formal documentation in hospital medical records, provided by a clinical geneticist. Acceptable evidence included karyotype analysis for chromosomal syndromes (e.g., trisomy 21, Turner), molecular or biochemical tests when available (e.g., gene sequencing for connective tissue disorders), or specialist reports recorded in the Genetics Department registry. Not all patients underwent identical testing, as the diagnostic approach varied by syndrome and clinical protocols at the time (2011–2014). However, all included cases had a definitive diagnosis issued by a certified clinical geneticist and registered in hospital records. Cases with only clinical suspicion but lacking documented genetic confirmation were excluded. Patients with unrelated dental pathology or comorbidities that could confound the analysis were exclude, as well. Of the 228 patients initially screened, 15 were excluded: 11 due to lack of documented genetic confirmation and 4 due to unrelated dental pathology (e.g., trauma, cleft lip/palate). The final analytic sample therefore comprised 213 patients.

Although no formal ethics approval number was issued at the time of original data collection, institutional access to records and registers was granted by the hospital’s internal board, in accordance with local clinical regulations. In 2021, the Ethics Committee of the Faculty of Medicine and Pharmacy, University of Oradea (Approval No. 27/31.03.2021) granted retrospective clearance specifically for the scientific reanalysis of these anonymized data. Informed consent had been obtained from legal guardians at the time of examination, and assent was obtained from patients when appropriate. All data were anonymized prior to analysis.

### 2.2. Clinical Dental Examination

Dental assessments were performed under standardized clinical conditions, using sterile dental mirrors, periodontal probes, and gauze, with appropriate illumination. Each participant’s dentition was thoroughly examined for anomalies in five categories: tooth number, size, shape, structure, and eruption pattern. Dental anomalies were recorded and classified according to the standardized morphological framework proposed by de La Dure-Molla et al. (2019) for genetic dental disorders [17]. In addition to this framework, we introduced a pragmatic grouping into “unique” versus “associated” anomalies, the latter including dyads (two anomalies) and triads (three anomalies). This supplementary categorisation, while not universally applied, was used to highlight the recurrent clinical patterns observed in syndromic patients and to facilitate the epidemiological analysis of co-occurring defects. For the purposes of this study, anomalies were grouped into five major categories: (1) number anomalies (hypodontia, oligodontia, hyperdontia); (2) size anomalies (generalized or localized microdontia, macrodontia); (3) form anomalies (taurodontism, double teeth, invaginated tooth, transposition); (4) structure anomalies (enamel hypoplasia, enamel hypomineralization, dentin defects); and (5) eruption anomalies (delayed eruption, impaction, ectopia, dilaceration, transposition).

Where a dental anomaly was suspected, existing radiographs were reviewed; new imaging was requested only when clinically justified, in accordance with radiation safety protocols. Clinical photographs were also taken to document visible anomalies, with full respect for anonymity and data protection regulations. Cases without available radiographs were assessed solely on clinical findings, and no patients were excluded due to missing imaging data. Thus, radiographic information was incorporated when present, but absence of imaging did not preclude inclusion in the analysis. Radiographic evaluation was based primarily on panoramic radiographs, as full-mouth series were rarely available in this age group. Clinical photographs were obtained in a standardized intraoral format (frontal, lateral, occlusal) whenever possible. For patients lacking photographs, findings were documented directly in clinical records. Thus, evaluations combined direct clinical notes with supplementary imaging.

All examinations were performed by a single trained examiner. Intra-examiner reproducibility was assessed by re-evaluating a subset of records after a two-week interval, showing >80% agreement. For the present retrospective analysis, all records and photographs were again reviewed by the same investigator to ensure consistency.

All examinations and data collection were performed under the supervision of hospital staff at the Genetics Department of Dr. Gavril Curteanu Municipal Hospital, Oradea. All procedures were conducted in accordance with clinical care standards and ethical norms. Data documentation and imaging were performed with full respect for patient anonymity, under institutional oversight and in line with the approved ethical framework.

The final sample represented all adolescents with genetically confirmed syndromes who met the inclusion criteria during the 2011–2014 collection period. Given the rarity of these conditions, no a priori sample size calculation was performed.

### 2.3. Statistical Analysis

Data were initially collected and organized in Microsoft Excel, where descriptive statistics were calculated for the frequency and distribution of dental anomalies across syndromes. Both absolute counts and relative prevalence were recorded for each anomaly category. To quantify associations between dental anomalies and specific syndromes, the rate of occurrence ratio (ROR) was employed. ROR was selected because the study objective was to assess disproportionality between observed and expected frequencies across multiple syndromes, rather than to model individual-level risk as in logistic regression or to compare predefined groups as in prevalence ratios. In this descriptive, cross-sectional context, ROR offered an appropriate way to highlight whether specific anomalies occurred more or less frequently than expected within the sample. In this context, ROR quantifies whether a specific anomaly or syndrome occurs more frequently (ROR > 1) or less frequently (ROR < 1) than expected relative to the rest of the cohort. ROR values were calculated only for syndromes with more than five confirmed cases in the study sample, to ensure statistical stability and interpretative relevance of the resulting estimates.

Data analysis was conducted using JASP (version 0.19.1). The Generic Effect Size module was used for meta-analytical computations. Log-transformed ROR values and their standard errors (SE) were derived using Woolf’s method. The resulting forest plot was automatically generated and exponentiated in JASP to reflect interpretable ROR values. 95% confidence intervals (CIs) were calculated, and only RORs whose CI did not include 1 were considered statistically significant.

Artificial intelligence (AI) tools were employed to assist in the linguistic refinement and clarity of the manuscript. Specifically, ChatGPT (version 5, OpenAI, San Francisco, CA, USA) was used to support English language editing and to improve grammar and phrasing without altering the scientific content.

## 3. Results

### 3.1. Patient Characteristics

A total of 213 adolescents aged 12–18 years with genetically confirmed syndromes were included in the study. The mean age of the study sample was 15.0 ± 1.8 years. The sex distribution was balanced, with 105 females (49.3%) and 108 males (50.7%), corresponding to a female-to-male ratio of approximately 1:1.03.

**Figure 1:**
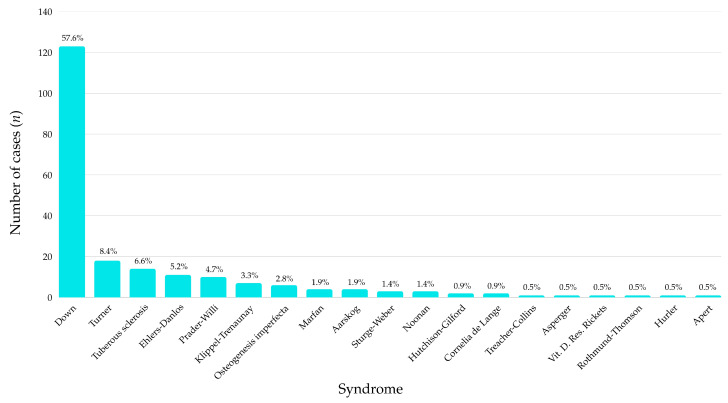
Prevalence of genetic syndromes in the pediatric study population (*n* = 213).

Among the 213 participants included in the study, Down syndrome was by far the most prevalent diagnosis, accounting for 57.6% of all cases (*n* = 123). This was followed at a considerable distance by Turner syndrome (8.4%) and tuberous sclerosis (6.6%). Several other syndromes, such as Ehlers-Danlos (5.2%), Prader–Willi (4.7%), and Klippel-Trenaunay (3.3%), were also represented in smaller but relevant proportions (Figure 1).

The remaining diagnostic categories, including Marfan, Aarskog, Sturge-Weber, Noonan, Hutchison-Gilford, and various rare conditions (e.g., Apert, Hurler, Rothmund-Thomson, vitamin D–resistant rickets) each accounted for fewer than 2% of the sample individually. Notably, a total of 18 distinct syndromes were identified, highlighting the genetic heterogeneity of the sample.

### 3.2. Overall Prevalence of Dental Anomalies

Among the 213 children and adolescents evaluated, 68.08% (*n* = 145) presented with at least one dental anomaly (Table 1). These were almost evenly divided between unique anomalies, affecting 34.74% of the sample (*n* = 74), and associated anomalies, found in 33.34% (*n* = 71).

The highest prevalence of anomalies was observed in patients with Down syndrome, where 80.49% (*n* = 99) presented with dental changes. Within this group, the distribution was nearly equal between unique (48 cases, 39.02%) and associated anomalies (51 cases, 41.46%). Several other syndromes demonstrated 100% prevalence, including Osteogenesis Imperfecta and Marfan syndrome.

In Osteogenesis Imperfecta, most anomalies were associated (83.33%), whereas in Marfan syndrome, all recorded anomalies were unique (100%). Syndromes such as Turner (27.78%), Ehlers-Danlos (27.27%), and Tuberous sclerosis (35.71%) showed relatively low prevalence of dental anomalies. Rare syndromes represented by single cases or with no anomalies (e.g., Hutchison–Gilford, Noonan, Klippel–Trenaunay, Treacher–Collins, Asperger, Rothmund–Thomson) are presented in Supplementary Table S1.

### 3.3. Patterns of Dental Anomalies

**Figure 2:**
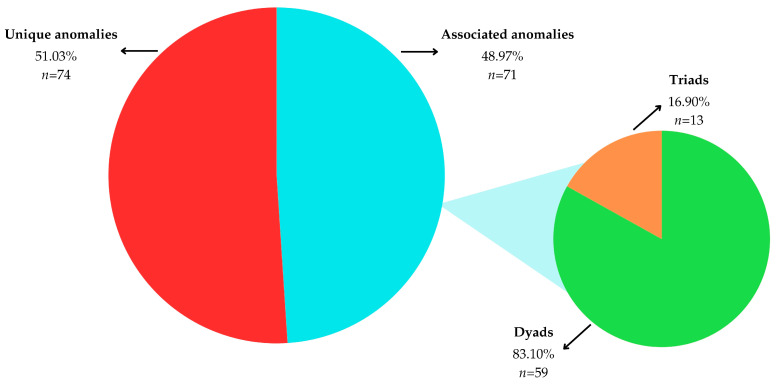
Distribution of unique and associated dental anomalies.

Out of the 145 cases with dental anomalies, 74 (51.03%) presented with unique anomalies, while 71 (48.97%) had associated anomalies involving combinations of two or more types (Table 2, Figure 2).

Within the unique anomaly group, form-related anomalies were most frequent, accounting for 28.38% of cases (9.86% prevalence in the full cohort). Anomalies of tooth number and tooth structure followed closely, each contributing 22.97% of unique anomalies (7.98% prevalence). Less common were anomalies involving eruption (13.51%) and tooth size (12.16%).

Among associated anomalies, dyads predominated. The most prevalent pairing was tooth number + form (40.85% of associated cases, 13.62% prevalence), followed by tooth number + size (30.99%, 10.33% prevalence). Rare dyads and complex triads occurred only in a small number of cases and are presented in Supplementary Table S2.

Among all documented cases, clinically relevant triads were less frequent, but included hypodontia + generalized microdontia + taurodontism (2.82% of the total sample) and generalized microdontia + dental inclusion + taurodontism (1.41%). These patterns were particularly observed in syndromes with a high dental burden such as Down syndrome and Osteogenesis Imperfecta, suggesting recurring developmental pathways with potential diagnostic value (Table 3).

Certain individual anomalies also stood out for their diagnostic relevance. Taurodontism was the most common unique anomaly (8.92%, *n* = 19), and in most cases it co-occurred with hypodontia. Generalized microdontia was identified in 4.23% of patients, while enamel hypoplasia appeared in 7.98%, either in isolation or as part of complex anomaly patterns.

Two-anomaly combinations were also common, particularly hypodontia + taurodontism (13.62%) and hypodontia + generalized microdontia (6.10%). These dyads again suggest a common developmental origin, likely linked to early disruptions in tooth bud formation and mineralization. Rare anomalies and combinations (≤2 cases), such as ectopic eruption, transpositions, or invaginated teeth, are presented in Supplementary Table S3.

### 3.4. Disproportionality Analysis (ROR)

To assess the strength of association between specific genetic syndromes and the presence of dental anomalies, a disproportionality analysis was performed using the Rate of Occurrence Ratio (ROR) with 95% confidence intervals.

The analysis revealed an elevated ROR for Down syndrome (ROR = 3.95, CI: 2.15–7.25), suggesting a higher likelihood of dental anomalies in these patients compared with the rest of the sample.

In contrast, several syndromes showed reduced ROR values below 1, including Turner syndrome (ROR = 0.15, CI: 0.05–0.44), Ehlers-Danlos (ROR = 0.16, CI: 0.04–0.62), Klippel-Trenaunay (ROR = 0.18, CI: 0.03–0.93), and Tuberous sclerosis (ROR = 0.23, CI: 0.08–0.73). Other syndromes, such as Prader–Willi (ROR = 0.69), Cornelia de Lange (ROR = 0.47), and Sturge-Weber (ROR = 0.94), also had ROR values below 1; however, their wide confidence intervals included 1, indicating a lack of statistical significance.

No syndrome other than Down syndrome showed a significantly elevated risk of dental anomalies.

**Figure 3:**
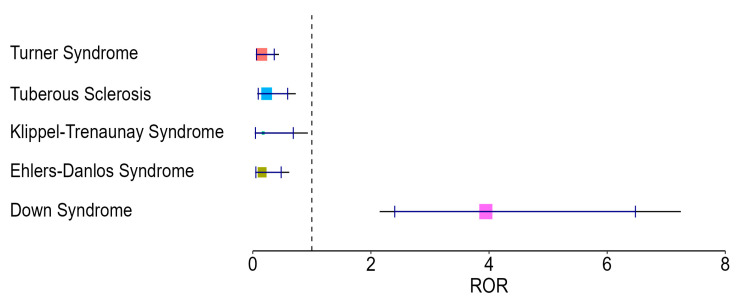
Graphical representation of Rate of Occurrence Ratio (ROR) with 95% confidence intervals for genetic syndromes significantly associated with dental anomalies. Legend: ROR values above 1 indicate a higher-than-expected prevalence of dental anomalies in the syndrome; values below 1 suggest lower prevalence compared to the rest of the cohort. Confidence intervals not overlapping 1 are considered statistically significant.

These findings are visually represented in Table 4 and Figure 3. Syndromes represented by fewer than five cases were excluded from ROR analysis to avoid unstable estimates; this exclusion should be considered when interpreting the findings.

## 4. Discussion

### 4.1. Patient Characteristics

The aim of this study was to analyze the prevalence and characteristics of dental anomalies in adolescents with genetically confirmed syndromes. Our findings confirm that such anomalies are highly prevalent, affecting more than two-thirds of patients, and that they follow non-random, syndrome-specific patterns with diagnostic relevance.

In this sample of adolescents aged 12–18 years, 68% presented with at least one dental anomaly, with some syndromes (e.g., osteogenesis-related or connective tissue disorders) showing 100% prevalence. These findings confirm previous reports that anomaly rates in syndromic populations far exceed those in the general population [17,18,19]. Down syndrome represented the majority of cases, reflecting not only its global frequency but also the greater likelihood of referral due to its easily recognisable phenotypic features [20,21]. Other syndromes, including Turner and tuberous sclerosis, were represented at intermediate frequencies and displayed oral-dental manifestations consistent with earlier studies [22,23]. The identification of rare conditions such as Apert, Hurler, and Rothmund-Thomson underscores the value of hospital and educational registries in capturing underdiagnosed disorders. Taken together, this distribution illustrates both the predominance of Down syndrome and the clinical relevance of rarer syndromes that may otherwise be overlooked without systematic screening.

### 4.2. Overall Prevalence of Dental Anomalies

More than two-thirds of the sample presented with dental anomalies, corroborating previous studies that highlight their clustering in syndromic populations, especially those with chromosomal or connective tissue disorders [20,21,22]. This high prevalence reflects early disruptions of ectodermal derivatives, often affecting teeth, skin, and craniofacial structures. Clinically, multiple or unusual anomalies may therefore serve as early diagnostic markers, warranting genetic evaluation and multidisciplinary follow-up.

The particularly high prevalence in Down syndrome and Osteogenesis Imperfecta aligns with their well-documented oral phenotypes [20,24,25]. By contrast, the absence of anomalies in conditions such as Treacher-Collins or Asperger syndrome may reflect distinct developmental pathways or the limited subgroup sizes in this study.

### 4.3. Patterns of Dental Anomalies

The distinction between unique and associated anomalies provides important clinical insights. In our cohort, form anomalies (e.g., taurodontism, peg-shaped teeth, transpositions) were the most frequent unique findings, followed by anomalies of tooth number and structure, in line with previous reports showing that morphodifferentiation is one of the earliest processes affected in syndromic dentition [26,27].

Nearly half of the affected patients presented with associated anomalies, most often the combination of hypodontia and altered tooth form. This dyad, well documented in Down syndrome and ectodermal dysplasias [28], reflects disruptions in both early dental lamina formation and later morphogenetic events. A more complex but characteristic triad, hypodontia, generalized microdontia, and taurodontism, was observed mainly in Down syndrome and Osteogenesis Imperfecta, and has also been described in ectodermal dysplasias and RASopathies [29,30]. These recurring patterns underline that syndromic dentition is shaped by coordinated disturbances across multiple stages of odontogenesis.

Although less frequent, anomalies like inclusion, ectopia, or transposition should not be overlooked, as they may provide diagnostic clues even in the absence of confirmed genetic testing. Taken together, these findings show that dental anomalies in syndromic patients occur in recurring, syndrome-specific patterns rather than as isolated defects. Recognizing these patterns is crucial for early diagnosis and tailored multidisciplinary care.

### 4.4. Disproportionality Analysis (ROR)

The disproportionality analysis indicated varying degrees of association between specific syndromes and dental anomalies. Down syndrome demonstrated an elevated ROR, consistent with previous reports of its characteristic oral phenotype [20,21,31,32]. In contrast, Turner syndrome, Ehlers-Danlos, Klippel-Trenaunay, and Tuberous sclerosis showed lower ROR values, which may suggest weaker associations in this sample, though these results should be interpreted with caution given the small sample sizes involved.

Syndromes such as Cornelia de Lange, Sturge-Weber, and Prader–Willi yielded wide confidence intervals due to small sample sizes, limiting interpretability. Nonetheless, these results may still offer a comparative framework for identifying conditions where dental screening is particularly informative.

### 4.5. Clinical Implications

Our findings have important implications for both dental specialists and general practitioners. Adolescents with genetic syndromes often present with complex dental anomalies that cluster in recognizable patterns, such as the frequent association of hypodontia and taurodontism. For general practitioners, awareness of these features is valuable: unusual dental traits observed during medical visits may serve as early markers of an underlying syndrome and should prompt timely referral for dental and genetic evaluation. For dental professionals, early interceptive measures, such as prosthetic replacement of missing teeth or protective management of enamel defects, can preserve function, guide jaw development, and reduce caries risk, ultimately improving quality of life and social integration [33]. Interdisciplinary planning, including early orthodontic assessment, is frequently required given the high prevalence of malocclusion in these populations [34,35,36]. Compared with non-syndromic patients, management in syndromic cases is more complex: anomalies are rarely isolated, often require long-term planning across specialties, and may be compounded by medical comorbidities, nutritional challenges, or behavioral difficulties [37]. This underscores the importance of multidisciplinary collaboration involving pediatricians, geneticists, psychologists, and dental specialists to provide coordinated care [38,39]. Nonetheless, many families continue to face barriers to adequate dental care, including limited access to trained providers, financial constraints, and perceived discrimination, underscoring the need for systemic policy changes [31].

### 4.6. Limitations and Strengths

This study has several limitations. First, its retrospective design relied on data originally collected between 2011 and 2014; although diagnostic classifications were updated for the present analysis, this time gap may affect applicability to current clinical populations. Second, uneven representation of syndromes was unavoidable: while the overall sample was relatively large, many individual conditions were represented by only a few patients, limiting the strength of syndrome-specific conclusions. Third, recruitment from a tertiary care center may have introduced referral bias, with more complex or severe cases likely overrepresented. This context may have led to an overrepresentation of dental anomalies compared with community-based populations. Fourth, no a priori sample size calculation was performed, as all eligible cases during the study period were included, which may limit the statistical power of subgroup analyses. In addition, all dental anomalies were recorded and verified by a single examiner, without independent cross-checking by another team member. The absence of inter-examiner calibration increases the possibility of observer bias. Finally, the use of multiple ROR comparisons introduces a potential risk of type I error, and the cross-sectional design without longitudinal follow-up restricted the ability to assess the onset, natural history, or progression of anomalies over time.

Despite these limitations, the study also has important strengths that balance potential weaknesses. First, it included a relatively large sample of adolescents with genetically confirmed syndromes, which is unusual given the rarity of these conditions. Second, dental anomalies were recorded using a standardized and widely recognized classification system, ensuring consistency and comparability. Third, the availability of both radiographic and photographic documentation enhanced diagnostic precision beyond clinical notes alone. In addition, focusing on adolescents (12–18 years) allowed the identification of anomalies that become fully apparent only once permanent dentition is established, while the systematic distinction between unique and associated anomalies highlighted clinically relevant patterns. Finally, the use of ROR-based disproportionality analysis provided a comparative perspective that extends beyond descriptive prevalence. Together, these strengths provide a solid basis for identifying syndrome-specific dental patterns and underline the value of integrating dental evaluation into multidisciplinary care for genetic syndromes.

## 5. Conclusions

Dental anomalies were identified in over two-thirds of adolescents with genetic syndromes, with taurodontism and hypodontia as the most consistent patterns. Focusing on adolescents allowed the detection of anomalies that become evident only in permanent dentition. Disproportionality analysis confirmed a strong association with Down syndrome, while other conditions showed lower frequencies. Overall, these findings suggest that dental anomalies may serve as useful indicators in syndromic evaluation and multidisciplinary care.

## Figures and Tables

**Table 1:** Prevalence of dental anomalies by type of genetic syndrome.

	Unique Anomalies		Associated Anomalies		Total Anomalies	
Genetic Syndrome	(*n*)	(%)	(*n*)	(%)	(*n*)	(%)
Down syndrome	48	39.02	51	41.46	99	80.49
Osteogenesis imperfecta	1	16.67	5	83.33	6	100
Turner syndrome	3	16.67	2	11.11	5	27.78
Ehlers-Danlos	0	0	3	27.27	3	27.27
Prader–Willi	4	40	2	20	6	60
Sturge-weber	1	33.33	1	33.33	2	66.67
Marfan syndrome	4	100	0	0	4	100
Tuberous sclerosis	5	35.71	0	0	4	35.71

**Table 2:** Distribution and prevalence of dental anomaly groups.

Dental Anomaly Group	Number of Cases (*n*)	% of Total Anomalies	Prevalence (%)
*Unique dental anomalies*	74	51.03	34.74
Number anomalies	17	22.97	7.98
Size anomalies	9	12.16	4.23
Structure anomalies	17	22.97	7.98
Eruption anomalies	10	13.51	4.69
Form anomalies	21	28.38	9.86
Associated dental anomalies	71	48.97	33.33
*Two combined anomalies*	59	83.10	27.70
Number + size	22	30.99	10.33
Number + form	29	40.85	13.62
Other dyads	8	11.27	3.76
*Three combined anomalies*	12	16.90	5.63
Total dental anomalies	145	100.00	68.08

**Table 3:** Distribution of specific dental anomaly combinations by type.

Anomaly Combination	No.	(%)
Unique dental anomalies		
Generalized microdontia	9	4.23
Enamel hypoplasia	17	7.98
Dental inclusion	5	2.35
Taurodontism	19	8.92
Other unique dental anomalies	9	4.23
Combinations of two anomalies		
Hypodontia + taurodontism	29	13.62
Hypodontia + generalized microdontia	13	6.1
Hypodontia + localized microdontia	8	3.76
Other dyads	8	3.76
Combinations of three anomalies		
Hypodontia + generalized microdontia + taurodontism	6	2.82
Generalized microdontia + dental inclusion + taurodontism	3	1.41
Other triads	3	1.41

**Table 4:** Disproportionality analysis (ROR) for genetic syndromes and associated dental anomalies.

Syndrome	ROR	CI 95%
Turner Syndrome	0.15	[0.05–0.44]
Tuberous Sclerosis	0.23	[0.08–0.73]
Klippel-Trenaunay	0.18	[0.03–0.93]
Ehlers-Danlos	0.16	[0.04–0.62]
Down Syndrome	3.95	[2.15–7.25]
Cornelia de Lange	0.47	[0.03–7.55]
Sturge-Weber	0.94	[0.08–10.52]
Prader–Willi	0.69	[0.19–2.53]

## Data Availability

The data presented in this study are available on request from the corresponding authors. The data are not publicly available due to privacy reasons.

## Supplementary Materials

The following supporting information can be downloaded at: https://www.mdpi.com/article/10.3390/jcm14207217/s1, Table S1: Prevalence of dental anomalies by type of genetic syndrome; Table S2: Distribution and prevalence of dental anomaly groups; Table S3: Distribution of specific dental anomaly combinations by type.

## Abbreviations

The following abbreviations are used in this manuscript:

ROR	Reporting odds ratio
CI	Confidence interval
TLA	Genetic syndromes caused by mutations in the RAS-MAPK pathway
