# Supplementary material for: Oral Manifestations in Adolescents with Genetic Syndromes: A Retrospective Cross-Sectional Study
Source: J Clin Med. 2025 Oct 13;14(20):7217. doi: 10.3390/jcm14207217 (PMC12565033; doi:10.3390/jcm14207217)
Supplement: Supplementary file 1 [file jcm-14-07217-s001.zip › jcm-3895323-supplementary.pdf]

Table S1. Prevalence of dental anomalies by type of genetic syndrome

	**Unique anomalies**		**Associated anomalies**		**Total anomalies**	
**Genetic Syndrome**	**(*n*)**	**(%)**	**(*n*)**	**(%)**	**(*n*)**	**(%)**
Down syndrome	48	39.02	51	41.46	99	80.49
Osteogenesis imperfecta	1	16.67	5	83.33	6	100
Turner syndrome	3	16.67	2	11.11	5	27.78
Hutchison-Gilford	0	0	2	100	2	100
Ehlers-Danlos	0	0	3	27.27	3	27.27
Prader-Willi	4	40	2	20	6	60
Noonan syndrome	1	33.33	2	66.67	3	100
Aarskog syndrome	2	50	2	50	4	100
Sturge-weber	1	33.33	1	33.33	2	66.67
Marfan syndrome	4	100	0	0	4	100
Tuberous sclerosis	5	35.71	0	0	4	35.71
Klippel-Trenaunay	2	28.57	0	0	2	28.57
Treacher-Collins	0	0	0	0	0	0
Asperger	0	0	0	0	0	0
Cornelia de Lange	1	50	0	0	1	50
Vitamin D resistant rickets	1	100	0	0	1	100
Rothmund-Thomson	0	0	0	0	0	0
Hurler syndrome	0	0	1	100	1	100
Apert syndrome	1	100	0	0	1	100

Table S2. Distribution and prevalence of dental anomaly groups

**Dental anomaly group**	**Number of cases (n)**	**% of Total anomalies**	**Prevalence (%)**
Unique dental anomalies	74	51.03	34.74
Number anomalies	17	22.97	7.98
Size anomalies	9	12.16	4.23
Structure anomalies	17	22.97	7.98
Eruption anomalies	10	13.51	4.69
Form anomalies	21	28.38	9.86
Associated dental anomalies	71	48.97	33.33
Two combined anomalies	59	83.10	27.70
Number + size	22	30.99	10.33
Number + structure	4	5.63	1.88
Number + eruption	2	2.82	0.94
Number + form	29	40.85	13.62
Size + structure	1	1.41	0.47
Size + form	1	1.41	0.47
Three combined anomalies	12	16.90	5.63
Number + size + form	7	9.86	3.29

Number + eruption + form	1	1.41	0.47
Size + eruption + form	3	4.23	1.41
Size + structure + eruption	1	1.41	0.47
Total dental anomalies	145	100.00	68.08

Table S3. Distribution of specific dental anomaly combinations by type

**Anomaly combination**	**No.**	**(%)**
Unique dental anomalies		
Generalized microdontia	9	4.23
Enamel hypomineralization	2	0.94
Enamel hypoplasia	17	7.98
Ectopia	3	1.41
Dental inclusion	5	2.35
Transposition	2	0.94
Taurodontism	19	8.92
Double teeth	1	0.47
Invaginated tooth	1	0.47
Combinations of two anomalies		
Hypodontia + taurodontism	29	13.62
Hypodontia + generalized microdontia	13	6.1
Hypodontia + localized microdontia	8	3.76
Oligodontia + localized microdontia	1	0.47
Hypodontia + enamel hypoplasia	3	1.41
Hyperdontia + dental inclusion	1	0.47
Hypodontia + dental inclusion	1	0.47
Localized microdontia + enamel hypoplasia	1	0.47
Localized microdontia + dilaceration	1	0.47
Combinations of three anomalies		
Hypodontia + generalized microdontia + taurodontism	6	2.82
Oligodontia + localized microdontia + taurodontism	1	0.47
Hypodontia + dental inclusion + taurodontism	1	0.47
Generalized microdontia + dental inclusion + taurodontism	3	1.41
Localized microdontia + enamel hypoplasia + dental inclusion	1	0.47
